# Reduced Antioxidant Potential of LDL Is Associated With Increased Susceptibility to LDL Peroxidation in Type II Diabetic Patients

**DOI:** 10.5812/ijem.5029

**Published:** 2012-09-30

**Authors:** Nivedita Singh, Neelima Singh, Sanjeev Kumar Singh, Ajay Kumar Singh, Deepak kafle, Navneet Agrawal

**Affiliations:** 1 Department of Biochemistry, GR Medical College, Gwalior, India

**Keywords:** Diabetes Mellitus, LDL Oxidation Sensitivity, Antioxidant Potential (AOP)

## Abstract

**Background:**

Type II diabetes mellitus is a complex heterogeneous group of metabolic conditions characterized by an increased level of blood glucose, due to impairment in insulin action and/or insulin secretion. Hyperglycemia is a major factor in the pathogenesis of atherosclerosis in diabetes. Oxidative modification of low density lipoprotein (LDL) is recognized as one of the major processes involved in the early stages of atherosclerosis in type II diabetes. LDL contains different antioxidants, which increase LDL resistance against oxidative modification, this is known as its antioxidant potential (AOP).

**Objectives:**

The present study has been carried out to investigate the sensitivity of LDL to oxidation, AOP of LDL and to assess whether hyperglycemia in diabetes mellitus is associated with increased LDL oxidizability, and whether these relationships are related to diabetic complications.

**Patients and Methods:**

This study was carried out on 100 diabetic subjects, divided into two groups according to their glycosylated hemoglobin (HbA1c) values, either regulated ( < 0.50 M hexose/ M Hb) or unregulated ( > 0.50 M hexose/ M Hb.) A further 50 healthy subjects were included to determine the sensitivity of LDL oxidation and measurement of LDL AOP. LDL from the serum sample was precipitated by the heparin-citrate precipitation method. The LDL fractions were exposed to oxidation with copper sulphate and their sensitivity to oxidation was evaluated. AOP was measured by taking measurements from 30 subjects in each group.

**Results:**

The sensitivity of LDL oxidation was significantly higher in both diabetic groups compared to the control group. AOP was significantly decreased in all diabetic groups compared to the control group.

**Conclusions:**

In type II diabetes, the increased susceptibility of LDL to oxidation is related to hyperglycemia and low AOP.

## 1. Background

Type II diabetes mellitus (DM) is a metabolic disorder characterized by hyperglycemia and insufficiency of secretion or action of endogenous insulin (1). Hyperglycemia plays an important role in the etiology of vascular complications such as atherosclerosis in diabetes, which is a major cause of morbidity and mortality in these patients (2). Cardiovascular disease (CVD), particularly coronary heart disease (CHD), is a major complication of this disease, and over 50% of all patients die of CHD. The most common cause of CHD is atherosclerosis (hardening of the arteries), where fatty deposits including cholesterol and other fats carried in the blood, build up as plaque on the inside of the artery wall. In the formation of plaque the peroxidation of lipoprotein, especially low density lipoprotein (LDL) plays a significant role (3).

LDL is one of the five major groups of lipoproteins, in size order, from largest to smallest these are; chylomicrons, very low density lipoproteins (VLDL), LDL, intermediate density lipoproteins (IDL), and high density lipoproteins HDL, which enable lipids such as cholesterol and triglycerides to be transported within the water based blood stream. LDL particles are the main carriers of cholesterol in the circulation and play a key role in cholesterol transfer and metabolism. Each native LDL particle contains a single apolipoprotein B-100 molecule (Apo B-100) and a highly hydrophobic core consisting of; polyunsaturated fatty acids, esterified cholesterol molecules and triglycerides. This core is surrounded by a shell of phospholipids and unesterified cholesterol. LDL particles are also rich in antioxidants such as; α-tocopherol, β-carotene and ubiquinol-10, which protects LDL from free radical attack and oxidation (4). Oxidation of LDL appears to be involved in the development of atherosclerosis (5). LDL oxidation leads to the alteration of LDL apolipoprotein-B (Apo-B) recognition sites and in the unregulated uptake of LDL by macrophages via scavenger receptors. The subsequent accumulation of cholesterol loaded macrophages (foam cells) in the subendothelium leads to the formation of fatty streak and atherosclerotic plaques. During LDL oxidation, three phases occur; lag phase, propagation phase and decomposition phase (6). In the lag phase, little oxidation occurs due to antioxidant defense mechanisms and the presence of antioxidant molecules. At the end of the lag phase, an antioxidant property of LDL is diminished and polyunsaturated fatty acids (PUFA) in LDL particles are rapidly oxidized to lipid hydroperoxides. After this propagation phase unstable lipid hydroperoxide starts to decompose and lipid peroxide concentrations decrease in the decomposition phase. LDL particles rich in antioxidants are more resistant to oxidation and have a longer lag phase in the oxidation process (7). LDL particles with poor antioxidant capacity have a shorter lag phase, therefore, the capacity of antioxidants present in the LDL which protect the LDL from oxidation by free radicals, is known as its antioxidant potential (AOP).

## 2. Objectives

This study was aimed at increasing our understanding of the effect of persisting hyperglycemia in type II diabetes, on the sensitivity of LDL oxidation, and LDL AOP.

## 3. Patients and Methods

The study was conducted on 150 human subjects, 50 of whom were normal and healthy (control group) and 100 who were suffering from type II diabetes mellitus. The diabetic patients were selected from the Intensive Care Unit (Medicine Department) and Medicine OPD, J.A. Group of Hospitals, Gwalior, India. The type II diabetic patients were divided into two groups; group 1 were regulated diabetic patients without complications (50 diabetic patients), and group 2 were unregulated diabetic patients with complications (50 diabetic patients). Complications in the diabetic patients included coronary artery disease which was diagnosed by clinical symptoms of angina pectoris, electrocardiogram examination or documented myocardial infarction. Glycosylated hemoglobin (HbA1c) values were used to categorize the diabetic groups. In our laboratory the reference interval has been established as 0.25-0.28 M hexose/M Hb healthy individuals, 0.28-0.50 M hexose/M Hb patients with regulated diabetes and > 0.50 M hexose/M Hb unregulated diabetes. All diabetic patients received oral hypoglycemic agents such as sulphonylureas or metformin. Diabetic patients with coronary artery disease were treated with calcium antagonists.

All of the subjects were between 50-60 years of age. Age matched control healthy subjects were selected from known families. All ethical measures and the patients’ written consent were taken before starting the study. The blood samples were collected with the permission of the Professor and Head, of the Department of Medicine. A sample of approximately 10 ml of fasting venous blood was taken from each subject, between 9.00 and 11.00 am following a fast from 10.00 pm the previous day. Samples were stored at 4º C for immediate analysis and at -20º C for later analysis. The following assays were carried out on sample specimens of the patients and controls: Glycosylated hemoglobin (HbA1c) levels were determined by the Rai KB, Pattabiraman TN method (8). Fasting blood sugar and lipid profile (total cholesterol and HDL cholesterol) were measured by standard kits on an autoanalyser (Biosystems S.A. Barcelona). LDL and VLDL lipid profiles were calculated by the Friedewald formula (9).

### 3.1. Determination of LDL Oxidation Sensitivity

LDL oxidation was determined by measuring malondialdehyde (MDA) in precipitated LDL. MDA levels were used as an indicator of free radical generation, which was increased at the end of lipid peroxidation. LDL was precipitated by the heparin citrate precipitation method (10). In brief, 5 ml 0.064 M sodium citrate buffer, pH 5.04 with 50000 IU/l heparin, was mixed with 0.5 ml of serum, vertexed and centrifuged at 1000 g for 10 minutes. The supernatant was removed and the LDL precipitate was dissolved in 1ml 1% Triton X-100. Then the MDA level was determined (11). This is the basal MDA level. The MDA level was determined again using the same method in copper induced LDL samples. In this process, LDL samples were incubated at 37˚C with copper sulphate (CuSO4) 1mM. The difference between induced and basal MDA levels was used to evaluate the samples’ sensitivity to oxidation, and the results were expressed as nmol/mL (12).

### 3.2. Determination of Antioxidant Potential

Thirty regulated diabetic patients, 30 unregulated diabetic patients and 30 healthy controls were selected from the samples described above. Thirty patients from the unregulated group who had the highest HbA1c levels, 30 subjects from the regulated group who had the lowest HbA1c levels and 30 subjects who were randomly chosen from the control group with normal HbA1c levels. For the measurement of AOP, LDL samples were incubated with the xanthine-xanthine oxidase system in the presence of cod liver oil. After 1h incubation, MDA levels were measured in all samples (13).

### 3.3. Statistical Analysis

Data were analysed using Sigma Plot 2000 and expressed as mean ± SD. Significance of the values was calculated by one way analysis of variance (ANOVA) and a post-hoc Dunnett T3 test. For antioxidant potential values, a Student’s t-test was used.

## 4. Results

In the present study, significant differences were found between the diabetic groups and the controls, fasting blood glucose and HbA1c levels were high in both the regulated and unregulated diabetic groups (Table 1). However, the highest glucose concentrations were apparent in the unregulated diabetic group associated with complications. In our study, HDL-C levels were significantly decreased in unregulated diabetics, compared to both the regulated and control subjects (Table 1). TG levels were also significantly changed in unregulated and regulated diabetics when compared to the controls (Table 1). LDL-C levels were significantly higher in the unregulated diabetic subjects, compared to the controls (Table 2). In this study the sensitivity of LDL to oxidation was significantly higher in both of the diabetic groups, compared to the control subjects (Table 3). However, the AOP value was significantly decreased in both of the diabetic groups, compared to the controls (Table 3), but LDL oxidation sensitivity and AOP were significantly changed in unregulated diabetics, compared to the regulated diabetic groups (Table 3).

**Table 1:** Characteristics of the Subjects Included in the Study a

	Control	Type II Diabetes Mellitus	
		Regulated (Group I)	Unregulated (Group II)
Number	50	50	50
M/F ratio	26/24	22/28	29/21
Age, y	51.09 ± 9.05	58.45 ± 8.22	56.12 ± 6.89
BMI b, Kg/m^2^	24.87 ± 1.23	25.54 ± 1.65	26.89 ± 1.98
Duration, y	-	5.65 ± 2.36	9.76 ± 3.56
SBP b, mmHg	115 ± 5.02	120 ± 5.38	130 ± 5.08
DBP b, mmHg	78 ± 4.30	80 ± 4.10	84 ± 4.15

^a^Values are Means ± SD

^b^Abbreviations: BMI, Body Mass Index; SBP, Systolic Blood Pressure; DBP, Dystolic Blood Pressure

**Table 2:** Status of Biochemical Parameters in Diabetic and Control Subjects a

	Control (n = 50)	Type II Diabetes Mellitus	
		Regulated (n = 50) (Group I)	Unregulated (n = 50) (Group II)
**HbA1c**, M hexose/M Hb	0.26 ± 0.016	0.50 ± 0.04 ^b^	0.61 ± 0.05 ^b^
**Fasting blood sugar**, mg/dL	73.59 ± 9.24	118.06 ± 8.75 ^c^	158.45 ± 8.74 ^b^
**Total Cholesterol**, mg/dL	155.10 ± 16.78	167.33 ± 15.54 ^d^	198.50 ± 15.34 ^b^
**Triglycerides**, mg/dL	125.90 ±.23.54	153.33 ± 20.33 ^c^	184.67 ± 22.16 ^b^
**LDL ^e^ -Cholesterol**, mg/dL	81.45 ± 25.04	87.56 ± 24.15 ^d^	118.65 ± 20.54 ^b^
**HDL ^e^ -Cholesterol**, mg/dL	49.56 ± 8.28	45.67 ± 3.54 ^d^	32.17 ± 4.12 ^b^
**VLDL-Cholesterol**, mg/dL	25.95 ± 4.73	31.66 ± 4.32 ^c^	37.34 ± 12.15 ^b^

^a^Values Expressed as Mean ±SD

^b^P < 0.001

^c^P < 0.05

^d^NS- Non Significant

^e^Abbreviations: HDL-C, High density lipoprotein-cholesterol; LDL-C, Low density lipoprotein-cholesterol

**Table 3:** Status of LDL Oxidation Sensitivity and Antioxidant Potential (AOP) of LDL in Diabetic and Control Groups a

	Control	Diabetes Mellitus	
		Regulated (Group I)	Unregulated (Group II) (n = 50)
Sensitivity of LDL oxidation, nmol/ml (induced MDA-basel MDA)	1.76 ± 0.82 (n=50)	2.54 ± 2.15^c^ (n=50)	4.75 ± 3.05^b^ (n=50)
AOP of LDL, nmol/ml.h	2.56 ± 0.89 (n=30)	1.85 ± 0.72 ^c^ (n=30)	0.71 ± 0.41 ^b^ (n=30)

^a^Values expressed as mean ± SD

^b^P < 0.001

^c^P < 0.05

## 5. Discussion

Diabetes mellitus is a widespread disease which creates a significant social impact. DM is a syndrome characterized by abnormal insulin secretion, derangement in carbohydrate and lipid metabolism, and it is diagnosed by the presence of hyperglycemia. Hyperglycemia, a hallmark of the diabetic condition, depletes natural antioxidants and facilitates the production of reactive oxygen species (ROS) and induces oxidative stress. Thus, increases in ROS and impaired antioxidant defense contribute to the initiation and progression of micro- and macrovascular complications in diabetes (14, 15). Lipid alterations and oxidizability of lipoproteins have also been considered as contributory factors to oxidative stress in DM (16). In this study, sensitivity to LDL oxidation was significantly higher in all of the diabetics when compared with the control subjects. Possible reasons as described earlier, may also include the turnover time of the oxidative process. In the process of lipid peroxidation, initial antioxidant components of LDL (such as α, δ-tocopherol and ubiquinone-10) are consumed to overcome oxidative stress, so there is no end-product of lipid peroxidation during the lag phase. Normally antioxidants increase the lag time of lipid peroxidation. Therefore, the sensitivity of LDL oxidation is also dependent on its antioxidant contents, ie, antioxidant potential (AOP). In diabetes the lag phase of lipid peroxidation and α-tocopherol levels have been reported to be significantly reduced due to increase oxidative stress (17). Hence, decreased lag time due to low AOP, induces LDL oxidation sensitivity in diabetes. In our study, increased LDL oxidizability and decreased AOP were observed in the diabetic patients, regardless of the type of diabetes and complications, which is in agreement with other studies (18, 19).

Very few studies have been performed comparing in vitro oxidation of LDL in relation to its antioxidant potential. Previous studies have provided evidence for the role of LDL glycation and its increased levels in vitro oxidizability (20). It is possible that poor glycemic control may account for the elevated LDL oxidizability in our diabetic patients. However, our findings showed that in diabetes, even in the absence of complications in regulated diabetics, depletion in antioxidant contents (low AOP) might also be responsible for the increased oxidizability of LDL. In the presence of complications in unregulated diabetics, lipid alterations were seen and could influence the susceptibility of LDL to oxidation. Diabetic patients with complications had higher TG, LDL-C, and lower HDL-C concentrations than regulated diabetics without complications and controls, which is in agreement with previous reports (21). Oxidation of LDL induces LDL-C accumulation (22, 23). High levels of cholesterol activate thrombocytes and cause the release of substances that activate phospholipase A2. Hence, accumulated arachidonic acid may be metabolized to leukotrienes, thromboxanes, prostaglandins and malondialdehyde. During this metabolism, oxygen radicals may be produced, and under insufficient antioxidant capacity, these radicals may also trigger lipid peroxidation, increasing the susceptibility of LDL to oxidation. Hypertriglyceridemia induces changes in LDL size and LDL oxidizability (24, 25). As HDL inhibits the oxidative modification of LDL (26), its reduction in diabetic patients could influence the susceptibility of LDL to oxidation.

In conclusion, the present study demonstrated increased lipid peroxidation and reduced antioxidant potential in favor of oxidative stress in both regulated, without complications and unregulated with complications, type II diabetes mellitus. Our findings showed that patients with diabetes had an increased sensitivity to LDL oxidation and decreased AOP due to a shortening of the lag phase, because the antioxidant molecules in LDL are consumed rapidly to overcome oxidative stress. The clinical approach of this study is that determination of LDL oxidation and its AOP helps in the prevention of vascular complications in diabetic subjects. Therefore, regular diet, improvement of glycemic control and antioxidant intake in diabetic patients may maintain antioxidant defenses and reduce oxidative stress.
